# Observations on the Endemic Pygmy Three-Toed Sloth, *Bradypus pygmaeus* of Isla Escudo de Veraguas, Panamá

**DOI:** 10.1371/journal.pone.0049854

**Published:** 2012-11-21

**Authors:** Sam Kaviar, Jakob Shockey, Peter Sundberg

**Affiliations:** The Evergreen State College, Olympia, Washington, United States of America; Monash University, Australia

## Abstract

Our objective was to ascertain the population status of the Pygmy Three-toed Sloth, *Bradypus pygmaeus*, an IUCN Critically Endangered species, on Isla Escudo de Veraguas, Panama. *Bradypus pygmaeus* are thought to be folivorous mangrove specialists; therefore we conducted a visual systematic survey of all 10 mangrove thickets on the island. The total mangrove habitat area was measured to be 1.67 ha, comprising 0.024% of the total island area. The population survey found low numbers of *B. pygmaeus* in the mangrove thickets and far lower numbers outside of them. The connectivity of subpopulations between these thickets on the island is not established, as *B. pygmaeus* movement data is still lacking. We found 79 individuals of *B. pygmaeus*; 70 were found in mangroves and 9 were observed just beyond the periphery of the mangroves in non-mangrove tree species. Low population number, habitat fragmentation and habitat loss could lead to inbreeding, a loss of genetic diversity, and extinction of *B. pygmaeus*.

## Introduction

The Pygmy Three-toed Sloth, *Bradypus pygmaeus*, was first described as a species in 2001 [1]. *Bradypus pygmaeus* is morphologically distinct from *Bradypus variegatus*, most obviously in their reduced body size, although genetic differentiation has not been shown [2]. *Bradypus pygmaeus* are found only on the 4.3 km^2^ island of Isla Escudo de Veraguas ( = Isla Escudo), 17.6 km off the Caribbean coast of Panama [1]. To date, researchers have only observed pygmy sloths in the red mangroves (*Rhizophora mangle*) of Isla Escudo’s tidal areas, leading to the working hypothesis of obligate red mangrove dietary specialization within the species [1].

Little research has been conducted on *B. pygmaeus* [3]. The dwarfism of *B.pygmaeus* may be due to their folivorous dietary specialization [4], although this hypothesis is not entirely supported [2]. Alternatively, dwarfism may be the result of the inability of sloths to defend food territories [5]. *Bradypus pygmaeus* is an IUCN Critically Endangered species, and understanding the dietary needs of *B. pygmaeus* is critical to developing any conservation plan. Initially, we attempted to falsify the hypothesis that *B. pygmaeus* are obligate mangrove specialists by attempting to track individuals that were outside the mangroves and observe one eating from a non-mangrove tree. We spotted three sloths outside the mangroves within a few hours of arrival on the island but did not see them eat. For the next three weeks we searched for more *B. pygmaeus* outside the mangrove thickets but did not find any. The scarcity of individuals outside of mangrove thickets and the paucity of individuals within them spawned the current study ascertaining the population number of *B. pygmaeus.* Our research focused on assessing the total population size of *B. pygmaeus* and delineating the distribution of this population on Isla Escudo. We assessed mangrove habitat size and quality as well as geographic distribution of habitats on Isla Escudo.

## Methods

**Figure 1:**
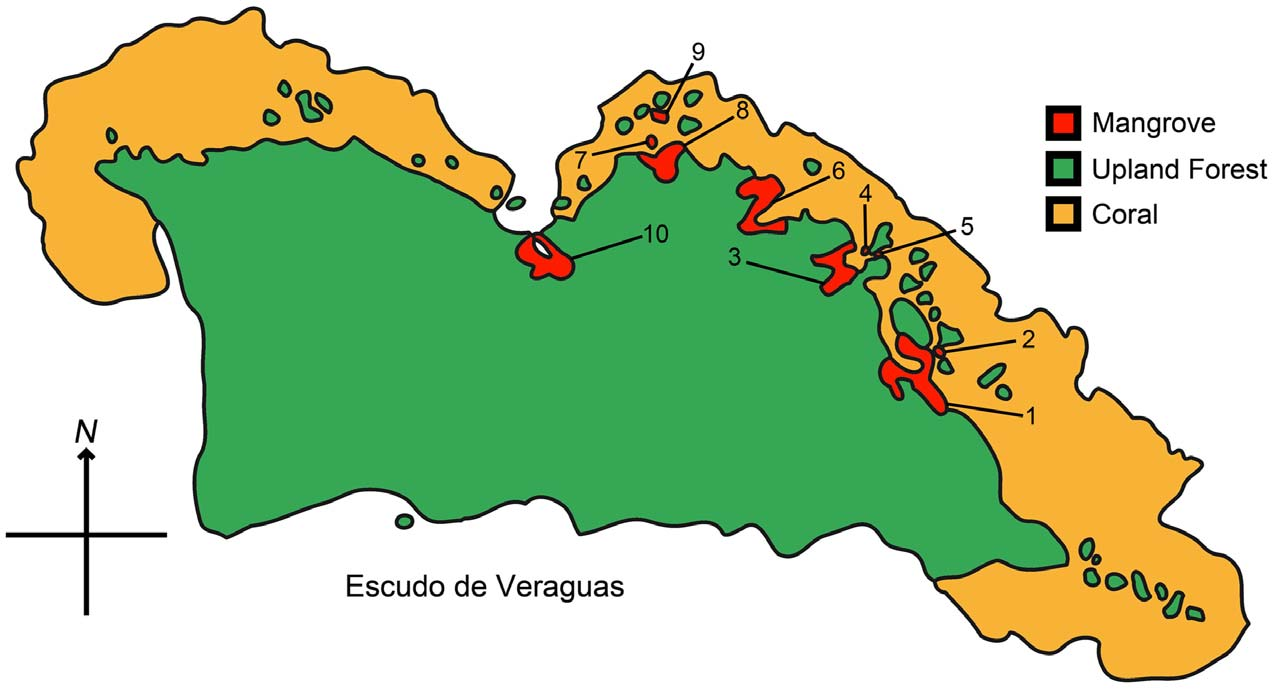
Map of all mangrove thicket locations found on Isla Escudo de Veraguas based on GPS data, showing thicket ID numbers.

**Figure 2:**
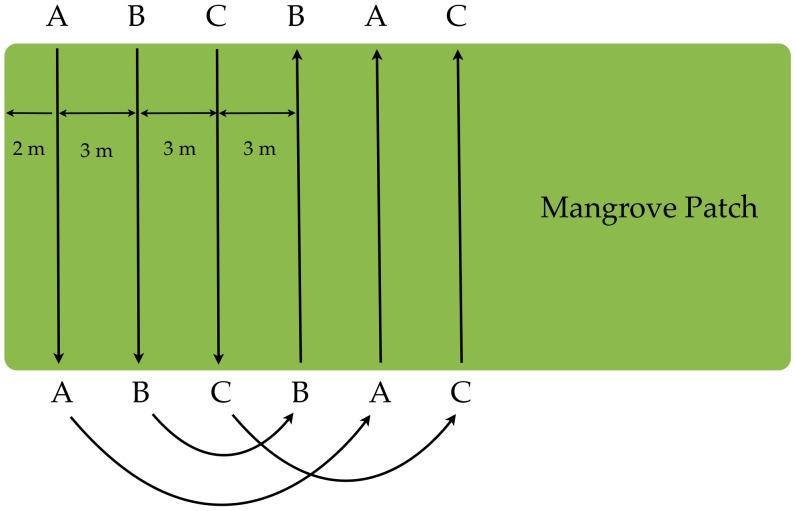
Schematic of strip census method used to survey mangrove thickets for *Bradypus pygmaeus*. Surveyors A, B, and C alternated positions for every survey strip.

We initially determined the location of all mangrove thickets and then surveyed them for sloths. First, we mapped the boundary of every mangrove thicket on Isla Escudo with Global Positioning System receiver (Garmin60CSx) waypoint tracks. We used ecological boundaries between mangrove species and other tree types as well as breaks in the canopy layer to define mangrove thickets, which we assigned ID numbers 1–10 (Fig. 1). After delineating each mangrove thicket, we conducted a line transect survey, with each of three observers spaced 3 m apart and walking a fixed compass bearing across the thicket. Each observer observed a strip width 4 m wide (2 m to each side). We did not control for canopy density in strip survey lines. When we reached far edge of the mangrove thicket the transect lines were shifted and the survey conducted along the opposite compass bearing. There was no overlap between transect lines (Fig. 2). This systematic visual survey was conducted throughout all mangrove thickets. When we encountered a sloth an identity number was assigned, and the sloth's location recorded with a GPS. Date, time, and notes on the physical appearance and dorsal coloration of each sloth were recorded. This strictly observational study was conducted under permit #SEA4311, titled "Status de *Bradypus pygmaeus* en Escudo de Veraguas, Panamá", from La Autoridad Nacional del Ambiente (ANAM). No animals were handled in this research.

This method was robust because the canopy density of mangroves is relatively thin, making for easy spotting of sloths. We also controlled for double counting by beginning and finishing the census for a thicket within the same day. Our census took place over a three-day time span. Mangrove thickets 1 and 2 were surveyed on May 8^th^, thickets 3–6 were surveyed on May 9^th^, and thickets 7–10 were surveyed on May 11^th^ 2011. We may have missed both sloths hidden in bromeliads on mangroves and babies that blended into their mother. Values are provided ±1 standard deviation.

**Table 1:** Results from census of *Rhizophora mangle* for *Bradypus pygmaeus*, Isla Escudo, 9° 5'58''N 81°33'22"W, May 2011.

Thicket	Area (m^2^)	Population (individual)	Density (ind./100 m^2^)
Thicket 1	35094	16	0.046
Thicket 10	23095	11	0.048
Thicket 3	18533	15	0.081
Thicket 6	14224	14	0.098
Thicket 8	13579	13	0.096
Thicket 4	606	0	0
Thicket 9	569	0	0
Thicket 7	483	1	0.21
Thicket 2	445	0	0
Thicket 5	71	0	0
Total	106699	70	0.067
Average ± Standard deviation.	10670±12282	7±7.3	0.058±0.066

**Figure 3:**
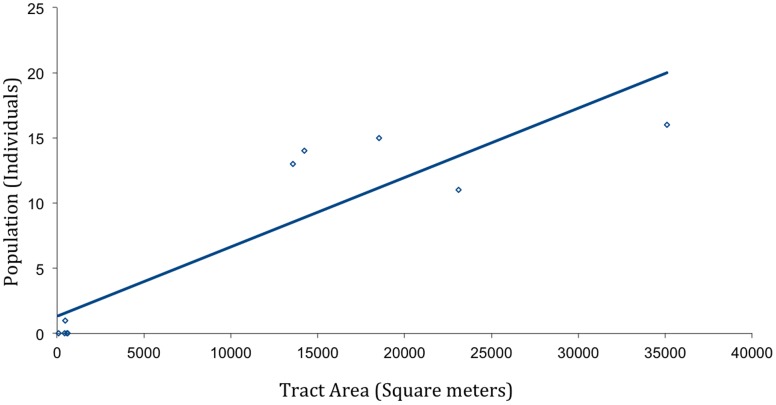
Regression analysis of population as dependent on (mangrove) area, where y = 0.0005×+1.3202 and R^2^ = 0.80493. The symbols for thickets 2,4 and 9 overlap.

## Results

We located 70 *B. pygmaeus* individuals within the mangrove habitat of Isla Escudo. This represents a minimum estimate, since we observed nine sloths outside of the survey areas. All of the sloths we observed in non-mangrove trees were within 20 meters of a mangrove thicket.

We mapped all mangrove stands upon Isla Escudo by GPS, from which data we generated a visual representation (Fig. 1). We calculate that the total mangrove habitat to be 106,699 m^2^, which is 0.024% of the total island area. Average mangrove thicket size was 10,670±12,282 m^2^ and the average presence of *B. pygmaeus* within a thicket was 7±7.3 individuals (Table 1). Densities of *B. pygmaeus* individuals were highest in the medium sized mangrove thickets (thickets 3, 6 and 8 at 0.081, 0.098 and 0.096 ind./100 m^2^ respectively), while the larger thickets (1 and 10) had lower densities (at 0.046 and 0.048 ind./100 m^2^ respectively). Nevertheless, in a simple linear regression (Fig. 3), these data suggest that overall population levels are dependent on mangrove thicket area, with y = 0.0005×+1.3202, and R^2^ = 0.80493.

## Discussion

It is critical for the survival of *B. pygmaeus* to ascertain whether or not they are obligate mangrove specialists and how able are they to disperse through the non-mangrove mixed lowland tropical forest, or utilize these forests for food. Our early fieldwork investigated whether any sloths found outside mangrove habitats consumed leaves. Although we spotted sloths in this non-mangrove forest at varies points in our fieldwork, we never saw one eat in this lowland forest. We saw sloths eating mangrove foliage on many occasions.

The mangroves on Isla Escudo are fragmented into five clumps separated by either non-mangrove, mixed forest or sea water. Within these thickets, we noted many instances of anthropogenic cutting of mangroves that interrupted the canopy layer between previously continuous mangrove habitat. The genus *Bradypus* is noted for its lack of ability to move on the ground [6]. Although we did not take data on the size or distribution of these cuts, we estimate them to occur over roughly 30% of the total mangrove habitat we surveyed. The distribution of this cutting between the ten identified mangrove thickets was not consistent, and may be a factor in *B. pygmaeus* density. We do not know the effects of these cuts on *B. pygmaeus*, yet we assume that this habitat disturbance and fragmentation could contribute to a decline of their population.

These deforested areas appear to be the result of logging by local people with hand tools. We observed numerous felled trees with machete and saw marks. It appears that the largest mangrove trees were selectively felled and in numerous thickets the largest mangroves trees we observed had been cut, but remained decomposing on the ground. Often the roots and branches of these trees were stripped away and the mangrove trunks were left behind. This observation supports the idea that mangrove logging takes place to support cooking fires using small diameter hardwood (Lenin Riquelme, Conservación, Naturaleza y Vida Panama (CONAVI), personal communication, October 21, 2010). In other thickets, the tallest standing mangrove trees were at the end of the thicket, farthest away from sea access.

We observed two dead *B. pygmaeus* bodies. The first carcass we found was decayed to bones and hair. We assume that these bodies were relatively fresh since decomposition happens quickly in the tropics; however, quantification of the time since death is not possible given our limited data on affecting environmental conditions. The second carcass was in a lesser state of decay, with some epidermal degradation, but overall recognizable features. These carcasses were still fully intact, suggesting that they were not killed by predation. If so, these observations lead us to suspect a high rate of death through disease, habitat loss, or natural causes in the population of *B. pygmaeus*. As an insular endemic species, *B. pygmaeus* may be adapted to a relatively high rate of inbreeding and have diminished risk of deadly recessive allele expression [7]. However, loss of allele variations could negatively affect the ability of *B. pygmaeus* to adapt to changes in its environment such as novel pathogens and climate change.

For *B. pygmaeus* to survive, protection of mangroves is needed. We also observed feral domestic cats on Isla Escudo, which is of concern to such a small *B. pygmaeus* population. Isla Escudo had four camps built of locally found materials in small deforested coastal areas. These camps house local Ngöbe fisherfolk who live in nearby mainland coastal communities but camp on Isla Escudo in order to have fishing trips at sea and around the island’s coral reefs. There is a camp on the eastern end of the island where fish was sold to fish buyers from Chiriquí Grande and elsewhere in Bocas Del Toro province for the local and international tourist market. The largest market was for lobster, however we observed many species of fish taken and one instance of shark finning in which the dorsal fins of five sharks were removed, and the sharks were left and died in the shallow breakwater of Isla Escudo. During our study we met with leaders of the Ngöbe community. It was brought to our attention that scientific research on Isla Escudo has been conducted without any discussion with the Ngöbe people. Conservation will fail if it does not include the local people [8].
