# Correction: Observations on the Endemic Pygmy Three-Toed Sloth, *Bradypus pygmaeus* of Isla Escudo de Veraguas, Panamá

**DOI:** 10.1371/annotation/ca64e77a-d4c4-4fc5-ab78-c284ae6e0606

**Published:** 2013-10-08

**Authors:** Sam Kaviar, Jakob Shockey, Peter Sundberg

There is an error in third sentence of the Abstract listing the total mangrove area. The correct sentence is: “The total mangrove habitat area was measured to be 10.67 ha, comprising 0.024% of the total island area.”

